# Temporal profiling of hippocampal astrocyte reactivity reveals persistent GFAP cleavage and complement C3 association after status epilepticus

**DOI:** 10.1007/s00429-026-03175-9

**Published:** 2026-08-07

**Authors:** Alisha Aroor, Zahra Sadri, Nicole Pinzón-Hoyos, Nicole D. Schartz, Amy L. Brewster

**Affiliations:** 1 Department of Psychological Sciences, Purdue University, West Lafayette, IN 47907 USA; 2 Department of Biological Sciences, Dedman College of Humanities and Sciences, Southern Methodist University, 6501 Airline Rd, DLSB 240; Lab DLSB 215/219, TX 75205 Dallas, USA

## Abstract

Astrocytes play essential roles in maintaining brain homeostasis, regulating synaptic transmission, supporting neuronal metabolism, and preserving blood-brain barrier integrity. Following prolonged seizures, such as status epilepticus (SE), astrocytes undergo structural and molecular changes that may contribute to epileptogenesis. Although reactive astrogliosis after SE is well documented, the temporal dynamics of astrocytic cytoskeletal remodeling and its relationship to complement activation remain incompletely defined. Using a pilocarpine-induced SE rat model, we examined hippocampal astrocyte responses at multiple time points (4 h, 1 day, 3 days, and 1-, 2-, 3-, or 5-weeks post-SE). Glial fibrillary acidic protein (GFAP) distribution was assessed by immunohistochemistry (IHC), and total GFAP protein levels and cleavage were quantified by western blotting (WB). We further evaluated the cellular association between GFAP-positive astrocytes and complement component C3 by immunofluorescence (IF). IHC revealed progressive astrocytic hypertrophy and increased GFAP immunoreactivity in the CA1 region, peaking at 2–3 weeks post-SE and modestly declining by 5 weeks. In contrast, WB demonstrated sustained elevations in total GFAP protein from 2 to 5 weeks post-SE, accompanied by significant accumulation of GFAP cleavage fragments. Quantitative analysis of GFAP breakdown products revealed progressive proteolytic processing during the latent-to-chronic phases of epileptogenesis. IF showed robust colocalization of C3 within hypertrophic GFAP-positive astrocytes at 2–3 weeks post-SE. When integrated with our prior mapping of microglial activation and complement dynamics, these findings indicate that astrocytic GFAP upregulation and cleavage follow early microglial activation and parallel sustained complement C3 expression. Together, our results demonstrate that SE induces dynamic and persistent astrocytic cytoskeletal remodeling characterized by coordinated GFAP synthesis and proteolysis, along with complement association. These findings highlight GFAP cleavage as a potential marker of astrocytic stress post-SE and support a temporally integrated astroglial-complement response following SE.

**Supplementary Information:**

The online version contains supplementary material available at https://doi.org/10.1007/s00429-026-03175-9.

## Introduction

Astrocytes are a type of glial cell characterized by complex, star-like processes that actively provide structural support and maintain brain homeostasis (Escartin et al. 2021). Under normal conditions, astrocytes are distributed throughout the brain parenchyma and perform specialized functions depending on their anatomical context. Astrocytes associated with the neurovascular unit (NVU) contribute to blood-brain barrier (BBB) integrity through their end-foot contacts with endothelial cells (Heithoff et al. 2021, Mills et al. 2022). In the parenchyma, astrocytes actively contribute to synaptic transmission and plasticity by regulating extracellular ion levels, clearing glutamate and GABA neurotransmitters at synapses, and taking up excess potassium to prevent excitotoxicity (Chung et al. 2015). Astrocytes can also produce and release gliotransmitters (e.g., ATP and D-serine) (Covelo and Araque 2018), recycle glutamate, and provide metabolic support by supplying lactate for neuronal energy (Beard et al. 2021). Given these multifunctional characteristics, astrocyte dysfunction is associated with the pathogenesis of many neurological conditions (Lee et al. 2022), including epilepsy (Vezzani et al. 2022, Bedner et al. 2024).

Following an epileptogenic insult, such as a prolonged and continuous seizure event, known as status epilepticus (SE), astrocytes undergo profound morphological, molecular, and functional alterations (Vezzani et al. 2022, Bedner et al. 2024). These changes include hypertrophy and proliferation (Shapiro et al. 2008, Borges et al. 2003, Arisi et al. 2011, Kim et al. 2008, Loewen et al. 2016, Estrada et al. 2012, Sano et al. 2021), upregulation of intermediate filament proteins such as those for glial fibrillary acidic protein (GFAP) (Takahashi et al. 2010, Schartz et al. 2023, Robel et al. 2015, Rossi et al. 2017), and altered expression of molecules needed for glutamate uptake and recycling (Coulter and Eid 2012), such as excitatory amino acid transporters (Hubbard et al. 2016, Peterson et al. 2021) and glutamine synthetase (Hammer et al. 2008, Sandhu et al. 2021). In epilepsy, these alterations have been linked to BBB instability, metabolic dysfunction, neuroinflammation, and the excitatory/inhibitory imbalance that contribute to spontaneous seizure generation (Vezzani et al. 2022, Bedner et al. 2024, Sandhu et al. 2021). Studies using rodent models of SE and acquired temporal lobe epilepsy (TLE) have demonstrated that astrocytic responses in the hippocampus are dynamic and time-dependent (Vezzani et al. 2022, Bedner et al. 2024, Shapiro et al. 2008, Borges et al. 2003, Arisi et al. 2011, Kim et al. 2008, Loewen et al. 2016). However, while increased GFAP expression is a hallmark of reactive astrogliosis, less is known about the temporal dynamics of astrocytic cytoskeletal remodeling, particularly GFAP proteolytic cleavage filament (Yang and Wang 2015), and how these changes align with inflammatory and complement-mediated processes during epileptogenesis.

Building on our prior work mapping microglia activation, neuronal degeneration, and complement signaling after SE (Schartz et al. 2024, Schartz et al. 2019, Schartz et al. 2018, Wyatt-Johnson et al. 2017, Schartz et al. 2016), we sought to determine how astrocytic GFAP alterations temporally align with these previously characterized changes within the same hippocampal tissues. We previously demonstrated that hippocampal microgliosis and inflammation peak during the subacute phase after SE (Wyatt-Johnson et al. 2017, Schartz et al. 2016), accompanied by dendritic degeneration and reduced MAP2 immunostaining in CA1 neurons (Sandhu et al. 2021, Schartz et al. 2016, Brewster et al. 2013), as well as sustained upregulation of complement components C1q and C3 (Schartz et al. 2019, Schartz et al. 2018). Microglial proliferation peaked at approximately two weeks post-SE and largely resolved by five weeks, when epilepsy was established (Schartz et al. 2018, Schartz et al. 2016).

The objective of the present study was to determine how astrocytic alterations relate temporally to these previously characterized events (Schartz et al. 2024, Schartz et al. 2019, Schartz et al. 2018, Schartz et al. 2016). To address this question, we used immunohistochemistry to assess GFAP spatial distribution, western blotting to quantify total GFAP protein levels, and evaluated GFAP proteolytic cleavage as an index of astrocytic cytoskeletal remodeling (Yang and Wang 2015), and Golgi staining to visualize astrocyte arborization, not fully captured by GFAP immunostaining alone (Yang and Wang 2015). This integrated approach allowed us to determine whether astrocytic GFAP upregulation and cleavage persist beyond microglial activation and parallel complement C3 expression, thereby providing a more comprehensive view of glial dynamics post-SE.

## Materials and methods

*Animals.* Adult male Sprague-Dawley rats (150–175 g; Envigo) were housed at the Psychological Sciences animal facility at Purdue University or the Laboratory Animal Research Core in the Dedman Life Sciences Building at Southern Methodist University. Rats were housed in pairs, maintained at 22 °C with a 12-hour light/dark cycle, and provided *ad libitum* access to food and water. All animal procedures were approved by the IACUC committees at Purdue University (Protocol #1309000927) or Southern Methodist University (Protocol #A21-003-BREA), following standard research guidelines (Percie du Sert et al. 2020).

*Pilocarpine-induced SE*. SE inductions were performed as previously described (Schartz et al. 2018, Schartz et al. 2016). Briefly, intraperitoneal (i.p.) injections of pilocarpine (280-300 mg/kg; i.p.) (Sigma-Aldrich, Cat. # P6503-10G) were administered 30 min after scopolamine methyl bromide (1 mg/kg, i.p.: Sigma-Aldrich, Cat. # S8502-1G). Controls received 0.9% saline (i.p.). Seizures were scored according to a modified Racine scale (Racine 1972) (1 = rigid posture, mouth moving; 2 = tail clonus; 3 = partial body clonus, head bobbing; 4 = rearing; 4.5 = severe whole body clonic seizures while retaining posture; 5 = rearing and falling; 6 = tonic-clonic seizure with jumping or loss of posture). Rats that reached stage ≥ 4.5 with continuous seizure activity for at least one hour were classified as severe SE and included in subsequent histological and biochemical analyses. Overall, about 75% of the rats treated with pilocarpine achieved and maintained a Racine scale score of 4.5 or higher, while 4–5% did not survive SE. After 1 h, SE was interrupted with Diazepam (10 mg/kg, i.p.; Hospira, Inc.). Following SE inductions, rats received 0.9% saline (i.p.) for hydration, and a supplementary diet of chocolate Ensure for up to 3 days post-SE (1 ml daily hand-fed). Brain tissues were collected at the following time points after SE: 4 h, 1 day, 3 days, and at 1-, 2-, 3-, or 5-weeks for biochemical analyses. For histology, the one-week time point was excluded. Histological and biochemical analyses were performed on tissues from different animal groups. Tissue collection for immunohistochemistry requires transcardial perfusion and tissue fixation to preserve morphology, whereas Western blot analysis requires rapid fresh tissue collection to preserve protein integrity.

*Immunohistochemistry (IHC).* IHC was performed exactly as previously described (Schartz et al. 2018, Schartz et al. 2016, Wyatt-Johnson et al. 2021). Briefly, animals were anesthetized with Beuthanasia (active ingredients: 390 mg pentobarbital sodium, 50 mg phenytoin sodium; 200 mg/kg, i.p) and transcardially perfused with ice-cold 1X Phosphate-buffered saline (PBS; 137 mM NaCl, 2.7 mM KCl, 4.3 mM Na2HPO4, 1.47 mM KH2PO4, pH 7.4) followed by 4% paraformaldehyde. Brains were postfixed overnight, cryoprotected in 30% sucrose, frozen, and stored at -80 °C until use. Serial coronal Sect.  (30 μm) were collected along the dorsoventral axis (Bregma − 3.00 mm and − 5.28 mm). Free-floating sections were immunostained with anti-mouse GFAP (1:1000; Cell Signaling, Cat. #3670) followed by biotinylated goat anti-mouse secondary antibodies (1:2000; Vector Laboratories, Cat. # BA-9200). Antibodies were diluted in goat immunobuffer (5% goat serum, 0.3% BSA, 0.3% PBS-T). Sections were incubated in an Avidin/Biotin complex solution (Vector Laboratories, Cat. # SP-2001) and developed with the DAB Substrate Kit (Cat. # SK-4100, Vector Labs). Development time and conditions were identical across all experimental groups. Sections were mounted on gelatin-coated slides, counterstained with Nissl stain, and cover-slipped using Permount mounting medium. Immunostaining was visualized using Leica DM5500/DM500 microscopes equipped with high-resolution digital cameras, or a Nikon Ti2-E fitted with a high-resolution digital camera (DS-Ri2 Color CMOS). The relative mean GFAP signal intensity was quantified bilaterally in 4–6 sections per brain using ImageJ V1.54p (NIH) by investigators blinded to treatment, as previously described (Schartz et al. 2018, Schartz et al. 2016). All chemicals were obtained from Fisher Scientific unless otherwise indicated. SE groups: 4 h (*n* = 4), 1 day (*n* = 3), 3 days (*n* = 5), 2 weeks (*n* = 6), or 5 weeks (*n* = 4); Controls (*n* = 5).

*Immunofluorescence (IF).* Brain sections were washed in 0.1% PBS-T, incubated in 3% PBS-T, and blocked with donkey immunobuffer (5% donkey serum, 0.3% BSA, 0.3% PBS-T) for 1 h at RT. Sections were then incubated overnight with the rabbit anti-C3 (1:1000; Abcam, Cat. # Ab200999). The following day, sections were washed in 0.1% PBS-T and incubated with Alexa Fluor 568 donkey anti-rabbit antibodies for 1 h at RT. Following washes, sections were incubated with mouse anti-GFAP Alexa Fluor 647 antibodies (Cell Signaling, Cat. #3657) and DAPI (Life Technologies, Cat. # D1306). Following washes, sections were mounted on slides, coverslipped with ProLong Gold antifade reagent (Invitrogen, Cat. # P36934). The IF signal was visualized and captured with a Nikon Ti2-E equipped with a high-resolution monochromatic digital camera. SE groups: 2 weeks (*n* = 5), 3 weeks (*n* = 5); Controls (*n* = 5).

*Western blotting (WB).* Protein homogenates were separated using standard SDS-PAGE protocols with 10% Tris-Glycine gels and transferred to PVDF membranes (Thermo Fisher Scientific, Cat. # 88518) for WB, as previously described (Schartz et al. 2024, Schartz et al. 2018). Briefly, membranes were blocked (5% milk in 1X PBS containing 0.1% Tween 20 (0.1% 1X PBST)) for 1 h at RT and incubated with mouse anti-GFAP antibodies (1:1000; Cell Signaling, Cat. #3670) overnight at 4 °C or for 2 h at RT. Rabbit anti-GAPDH antibodies (1:30,000; Millipore Sigma, Cat. # G9545) were used as the loading control. After washing with 0.1% 1X PBST, membranes were incubated for 1 h at RT with HRP-conjugated anti-mouse (1:1,000; Cell Signaling, Cat. #7076) or rabbit (1:1,000; Cell Signaling; Cat. #7074) secondary antibodies, washed, treated with ECL substrate (Thermo Fisher Scientific, Cat. # 32106), and developed on radiography film (MIDSCI, Cat. # BX57). Immunoreactive band signal intensities were quantified using Image J. All values were background-subtracted, normalized to the corresponding GAPDH band in the same lane, as previously described (Schartz et al. 2023, Schartz et al. 2019, Schartz et al. 2018). SE groups: 4 h (*n* = 3), 1 day (*n* = 7), 3 days (*n* = 7), 1 week (*n* = 4), 2 weeks (*n* = 10), 3 weeks (*n* = 10), or 5 weeks (*n* = 9); Controls (*n* = 14).

*Golgi staining.* Staining was performed using the FD Rapid Golgi Stain Kit (Neurodigitech, San Diego, CA) according to the manufacturer’s instructions. After perfusion, brains were rapidly removed and incubated in Golgi impregnation solutions for 4–6 weeks. Brains were then sectioned coronally at 80 μm, mounted on gelatin-coated slides, and stained according to the kit protocol. Sections were dehydrated through graded alcohol (50% to 100%), cleared in xylene, and coverslipped with Permount mounting medium. Images were acquired using either a Nikon Ti2-E with a DS-Ri2 Color CMOS camera or a Leica DM5500 equipped with a Leica DFC290 and LAS V4.6 software. Five representative sections along the dorsoventral axis (Bregma − 3.48 mm to − 5.28 mm) were analyzed. Group sizes: control (*n* = 3), 3 days post-SE (*n* = 3), 14 days post-SE (*n* = 3), and 35 days post-SE (*n* = 2) (Schartz et al. 2016).

*Statistical Analyses.* Statistical analyses were performed using GraphPad Prism. Normality was assessed using the Shapiro-Wilk test. As the data met assumptions of normality and homogeneity of variance, parametric tests were applied. Differences among experimental groups were analyzed using one-way ANOVA followed by Dunnett’s multiple-comparisons test. Homogeneity of variance was evaluated using the Brown-Forsythe and Bartlett’s tests. A p-value < 0.05 was considered statistically significant. Figures were assembled using Adobe Photoshop CS6.

## Results

SE triggers a change in GFAP-positive astrocytes in the hippocampus.

**Fig. 1:**
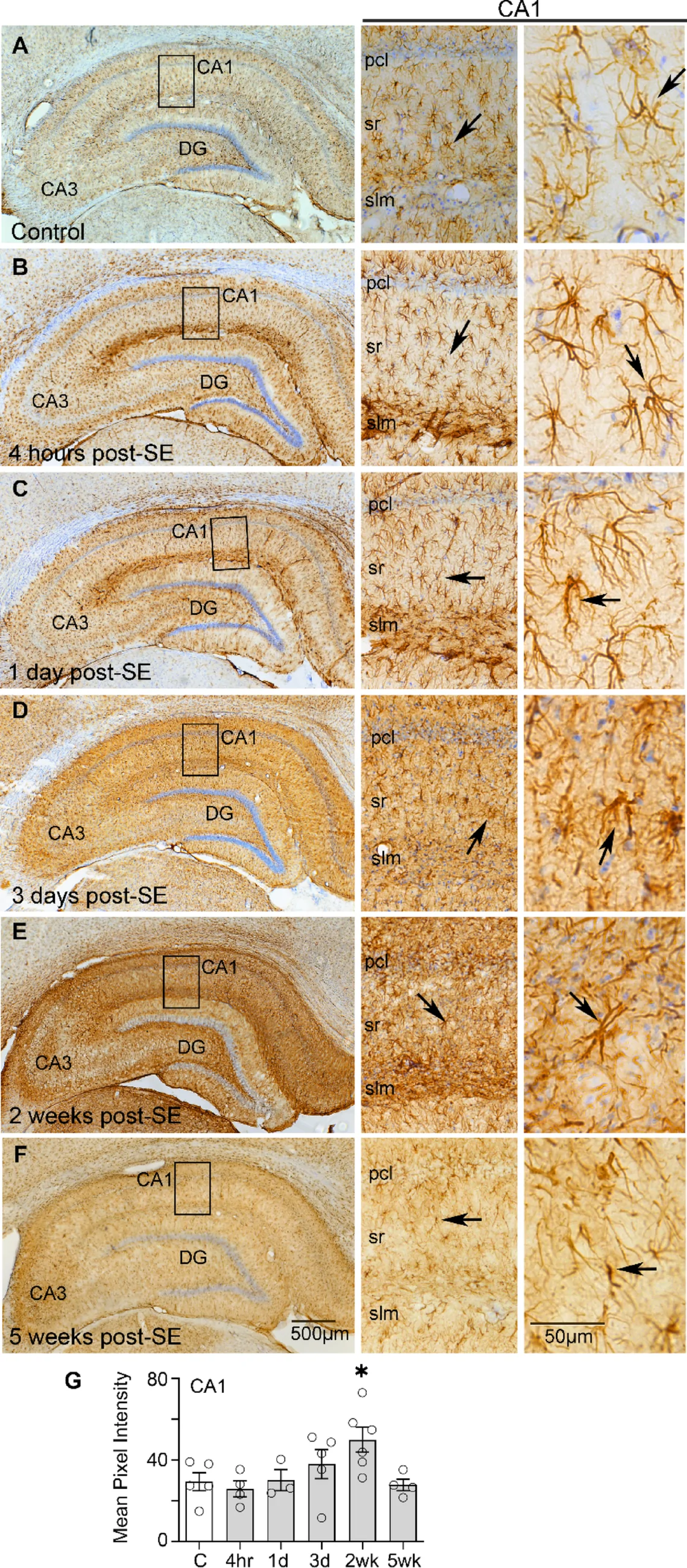
Temporal profile of GFAP immunostaining in the hippocampus after status epilepticus (SE). (**A**-**F**) Representative immunohistochemical images of hippocampal sections from control rats and at different time points after SE: (**A**) Control, (**B**) 4 h post-SE, (**C**) 1 day post-SE, (**D**) 3 days post-SE, (**E**) 2 weeks post-SE, and (**F**) 5 weeks post-SE. Low-magnification images (left panels) show the hippocampal subregions CA1, CA3, and dentate gyrus (DG). Black boxes indicate the CA1 region selected for higher-magnification analysis. The middle and right panels show enlarged views of the CA1 area. Arrows indicate astrocytes displaying immunopositive staining and morphological changes across time points after SE. Increased astrocytic staining intensity and hypertrophic morphology are evident at 3 days and 2 weeks post-SE, followed by a reduction at 5 weeks post-SE. Abbreviations: pcl, pyramidal cell layer; sr, stratum radiatum; slm, stratum lacunosum-moleculare. Scale bars: 500 μm (left panels) and 50 μm (middle and right panels). (**G**) Quantification of mean pixel intensity in the CA1 region across experimental groups: control (C), 4 h (hr), 1 day (1 d), 3 days (3 d), 2 weeks (2 wk), and 5 weeks (5 wk) post-SE. Data are presented as mean ± SEM. *p < 0.05 by one-way ANOVA with Dunnett’s post hoc test

We examined the distribution of GFAP-labeled astrocytes in hippocampi of control rats and the temporal progression of astrocyte alterations at 4 h, 1 day, 3 days, 2 weeks, and 5 weeks after a single episode of pilocarpine-induced SE (Fig. 1). Immunostaining showed a homogeneous distribution of GFAP-positive astrocytes throughout the hippocampal regions CA1, CA3, and dentate gyrus (DG) of control rats (Fig. 1A). Higher magnification images from the CA1 area revealed that astrocytes displayed characteristic morphological features, including stellate cell bodies with fine processes (Fig. 1A, arrows). SE triggered progressive changes in both the morphology of GFAP-stained astrocyte cell bodies and the density of their processes. At 4 h and 1 day after SE, astrocytic processes appeared thicker (Fig. 1B-C). Between 3 and 14 days post-SE, astrocytes exhibited hypertrophy, accompanied by strong immunoreactivity throughout the surrounding parenchyma (Fig. 1D-E). At 5 weeks, we observed a heterogeneous population of GFAP-positive astrocytes, including both hypertrophied cells and thin-process astrocytes (Fig. 1F). We measured the optical density of GFAP immunoreactivity in the CA1 region of the hippocampus (Fig. 1G). Densitometry analysis showed a peak in GFAP immunoreactivity 2 weeks after SE (*p* < 0.05). A one-way ANOVA demonstrated a statistically significant difference among the six groups, *F*(5, 21) = 3.06, *p* = 0.032. The Brown-Forsythe test for homogeneity of variances was not significant, *F*(5, 21) = 0.83, *p* = 0.541, indicating that the assumption of equal variances was met. Similar trends were observed in the CA3 (*F*(5, 23) = 2.00, *p* = 0.116) and dentate gyrus (*F*(5, 23) = 1.41, *p* = 0.258) regions, with no statistically significant differences among treatment groups (data not shown). Additional high-magnification images of the CA1, CA3, and DG regions are shown in Supplemental Fig. 1.

**Fig. 2:**
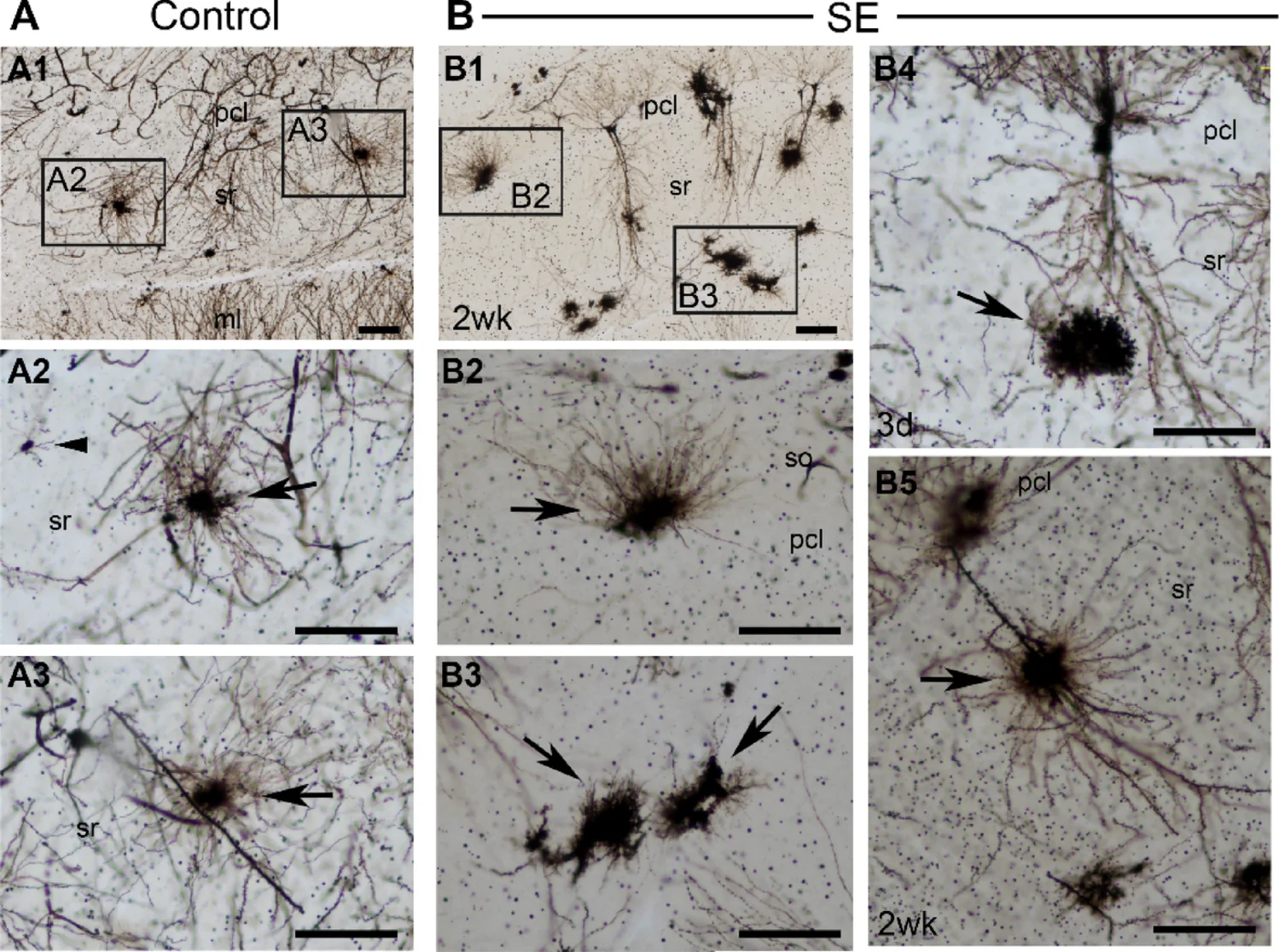
Golgi staining shows distinct astrocyte morphologies under control conditions and following status epilepticus (SE). (**A**) Control group. (**A1**) Low-magnification photomicrograph showing Golgi-stained astrocytes with typical morphology and distribution within the hippocampal layers. Boxed regions are enlarged in **A2** and **A3**. (**A2**-**A3**) Higher-magnification images of control astrocytes displaying small cell bodies and finely branched stellate processes (arrows). Arrowhead in A2 indicates a thin astrocytic process. (**B**) SE group. (**B1**) Low-magnification image obtained 2 weeks after status epilepticus (SE), showing astrocytes with altered morphology and increased process complexity. Boxed regions are enlarged in **B2** and **B3**. (**B2**) Higher-magnification image showing a hypertrophic astrocyte with thickened processes (arrow). (**B3**) Reactive astrocytes exhibiting enlarged somata and dense branching processes (arrows). (**B4**) Representative astrocyte 3 days after SE showing marked soma enlargement and process extension (arrow). (**B5**) Representative astrocyte 2 weeks after SE exhibiting pronounced reactive morphology with extensive arborization (arrow). Abbreviations: pcl, pyramidal cell layer; sr, stratum radiatum; ml, molecular layer; so, stratum oriens. Scale bars are indicated in each panel. Scale bars: low-magnification images (**A1**, **B1**) = 100 μm; high-magnification images (**A2**-**A3**, **B2**-**B5**) = 50 μm. These astrocytic morphologies were also present at 5 weeks post-SE (See Ref. (Schartz et al. 2016))

Because GFAP immunostaining does not capture the full extent of astrocyte arborization, which has been previously described and quantified in multiple studies (Yang and Wang 2015, Rossi et al. 2013), we used Golgi staining to visualize astrocyte morphology under control and SE conditions (Fig. 2). Note that Golgi staining sparsely and stochastically labels a subset of cells and is not specific to astrocytes, thereby enabling detailed visualization of individual cellular morphologies without labeling the entire astrocyte population. Astrocytes were identified using established morphological criteria in both the control and SE groups. In control samples, astrocytes exhibited thick somata with thin, elongated processes (Fig. 2A). In SE groups, astrocytes showed hypertrophic protoplasmic morphology, including enlarged somata, thickened processes, and expanded arbors (Fig. 2B2, B5), consistent with prior reports (Shapiro et al. 2008, Borges et al. 2003, Arisi et al. 2011, Kim et al. 2008). Clustered, bushy astrocytes with condensed, overlapping branchlets (Fig. 2B3) as well as astrocytes with truncated, beaded, or fragmented processes and swollen or vacuolized somata were also present (Fig. 2B4), as shown previously (Rossi et al. 2013, Kim et al. 2011, Kim et al. 2022).

**Fig. 3:**
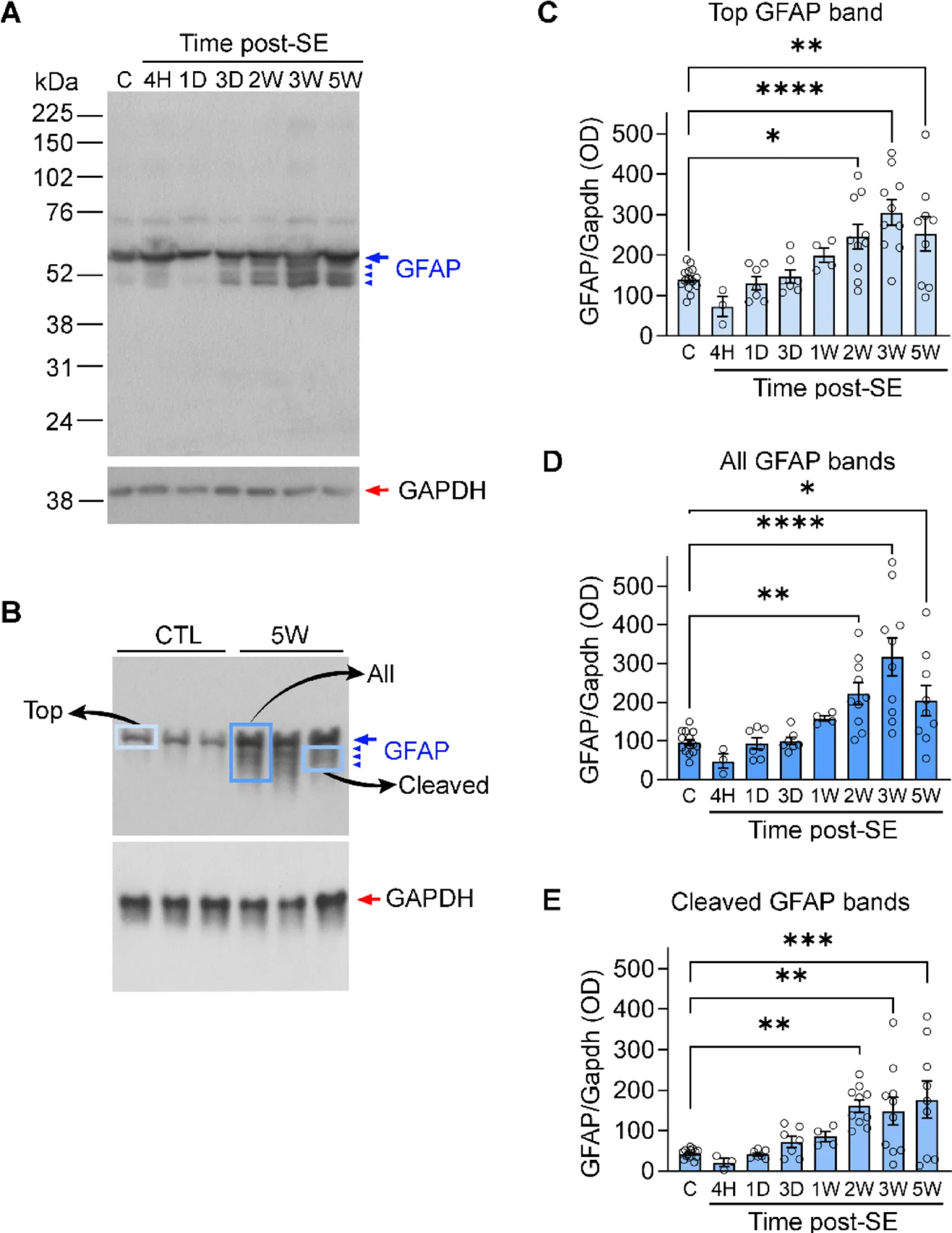
GFAP protein levels and cleavage are increased in the hippocampus following status epilepticus (SE). (**A**) Representative Western blot showing GFAP and loading control (GAPDH) levels in hippocampal whole homogenates from control rats (**C**) and at multiple time points after SE, including 4 h (4 H), 1 day (1D), 3 days (3D), 1 week (1 W), 2 weeks (2 W), 3 weeks (3 W), and 5 weeks (5 W) post-SE. The full-length GFAP (~ 50 kDa) protein is indicated by the blue arrow, and the proteolytic cleavage products are indicated by the arrowheads. (**B**) Quantification of GFAP protein levels normalized to GAPDH (red arrow) and shown as optical density (OD) (**C**-**E**). Molecular weight markers (kDa) are indicated on the left. Bars represent mean ± SEM; individual data points (circles) indicate biological replicates. * *p* < 0.05 by one-way ANOVA with Dunnett’s post hoc test

Next, we measured GFAP protein levels in whole hippocampal homogenates at 4 h, 1 day, 3 days, and at 1-, 2-, 3-, and 5-weeks after SE using immunoblots (Fig. 3 and Supplemental Fig. 2). Analysis of GFAP top-band immunoreactivity across experimental groups revealed a significant effect of time after SE (one-way ANOVA, *F*(7, 56) = 7.162, *p* < 0.0001) (Fig. 3A-C). Post-hoc comparisons using Dunnett’s test indicated that GFAP levels were significantly elevated compared to controls at 2 weeks (*p* = 0.0136), 3 weeks (*p* < 0.0001), and 5 weeks (*p* = 0.0092) post-SE. No significant differences were observed at 4 h, 1 day, 3 days, or 1 week after SE.

In addition, we observed cleavage of the GFAP protein into smaller fragments as early as 3 days after SE (Fig. 3A-B arrowheads), with cleavage becoming more pronounced at 2 to 5 weeks after SE, when spontaneous seizures begin in this rat model of SE and acquired TLE (Heithoff et al. 2021). GFAP can be cleaved at multiple sites by caspases, generating multiple fragments (Fig. 3B), which are thought to indicate its reactive state (Yang and Wang 2015). Analysis of all bands immunoreactive for GFAP showed a significant effect of time after SE (one-way ANOVA, *F*(7, 56) = 8.499, *p* < 0.0001) (Fig. 3A-C). Post-hoc comparisons using Dunnett’s test indicated that levels of all GFAP bands were significantly elevated compared to controls at 2 weeks (*p* = 0.0062), 3 weeks (*p* < 0.0001), and 5 weeks (*p* = 0.0335) post-SE. No significant differences were observed at 4 h, 1 day, 3 days, or 1 week after SE. Analysis of cleavage fragments showed a significant effect of time after SE (one-way ANOVA, *F*(7, 56) = 5.761, *p* < 0.0001) (Fig. 3A-C). Post-hoc comparisons using Dunnett’s test indicated that levels of all GFAP bands were significantly elevated compared to controls at 2 weeks (*p* = 0.0014), 3 weeks (*p* = 0.0052), and 5 weeks (*p* = 0.0004) post-SE. Together, these findings show that both the full-length (top-band) GFAP and its cleavage products increase over time following SE, particularly during the latent-to-chronic phases when spontaneous seizures emerge. The progressive increase in the main GFAP band indicates ongoing GFAP production, while the simultaneous buildup of cleaved GFAP fragments shows increased proteolytic activity linked to astrocyte reactivity (Yang and Wang 2015).

**Fig. 4:**
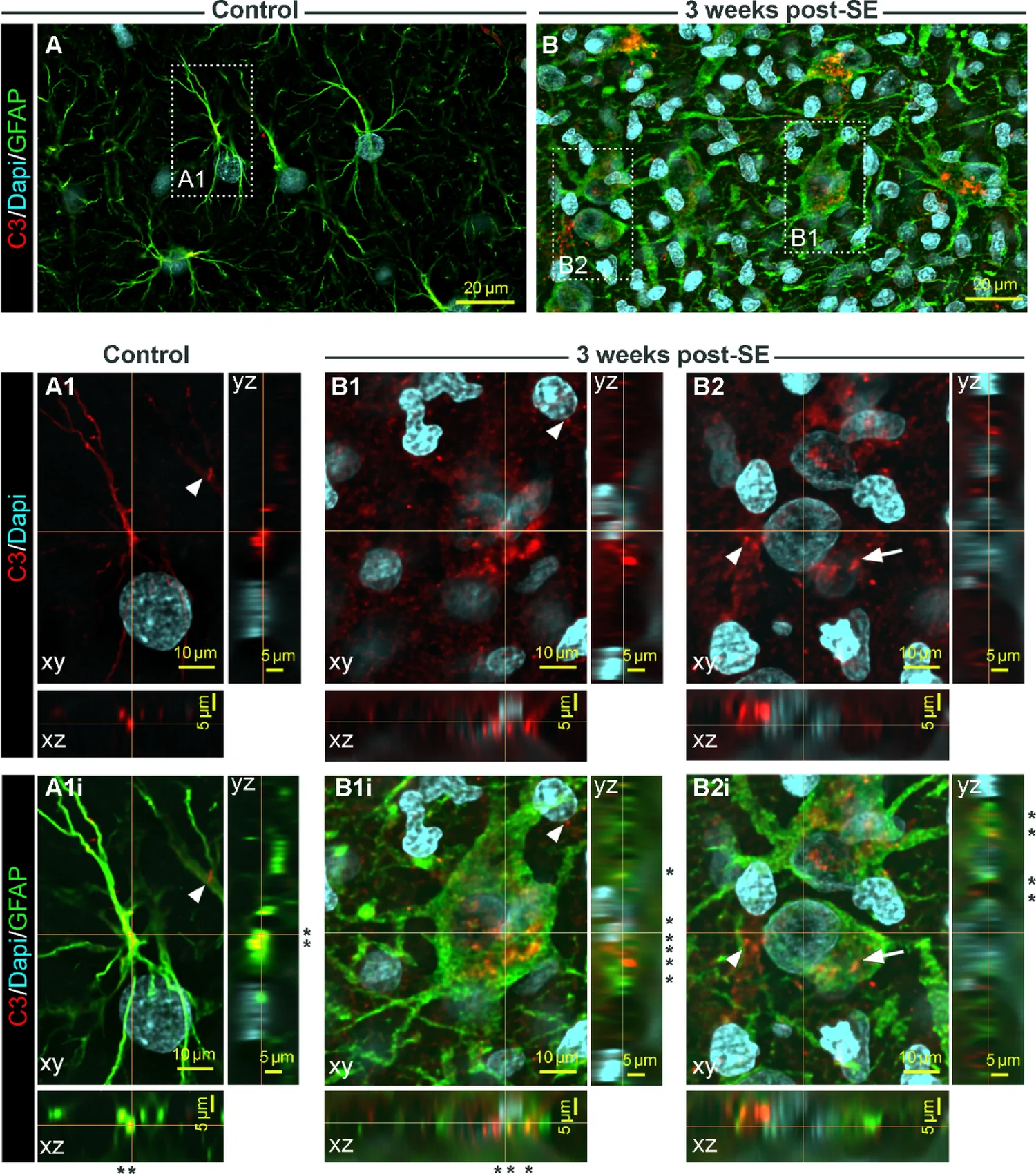
Reactive astrocytes accumulate complement component C3 following status epilepticus (SE). (**A**, **B**) Representative images of hippocampal tissue from control rats (**A**) and rats 3 weeks after SE induction (**B**). Sections were immunolabeled for GFAP (green), C3 (red), and DAPI-stained nuclei (blue). In control tissue, astrocytes displayed low basal C3 immunoreactivity, whereas prominent C3-positive puncta and diffuse labeling were observed at 3 weeks post-SE. Dashed boxes indicate regions shown at higher magnification in panels A1, B1, and B2. Scale bars: 20 μm. (**A1**, **B1**, **B2**) XY, XZ, and YZ image projections of the regions indicated above, showing C3 immunoreactivity (red) relative to cell nuclei (DAPI, cyan). In control tissue (**A1**), minimal C3 labeling was detected, while post-SE tissue (**B1**, **B2**) exhibited marked intracellular and pericellular C3 accumulation. Arrows indicate representative C3-positive structures that colocalize with GFAP, and arrowheads indicate C3 that does not colocalize with GFAP. Scale bars: xy, 10 μm; xz/yz, 5 μm. (**A1i**, **B1i**, **B2i**) Merged images including GFAP labeling (green) demonstrate localization of C3 immunoreactivity within GFAP-positive astrocytic processes and somata after SE. These image views confirm colocalization of the C3 signal with GFAP-labeled astrocytic structures. Asterisks denote regions of signal overlap. Scale bars: xy, 10 μm; xz/yz, 5 μm

Lastly, we compared the temporal trajectory of SE-induced GFAP changes in the hippocampus with previously reported alterations for microglia (IBA1) (Wyatt-Johnson et al. 2017, Schartz et al. 2016), dendrites (MAP2) (Schartz et al. 2016), and complement components (C1q and C3) (Schartz et al. 2024, Schartz et al. 2019, Schartz et al. 2018). SE-induced changes in GFAP protein levels closely mirrored the trajectory of C3, with sustained increases observed at 2 and 3 weeks post-SE (Schartz et al. 2018). Based on our previous findings demonstrating elevated C3 expression after SE (Schartz et al. 2018), and supported by a recent study in mice (Jeong et al. 2025), we further investigated the spatial association between GFAP and C3 at 2 and 3 weeks post-SE. Dual immunofluorescence labeling revealed limited colocalization of C3 with GFAP-positive astrocytes under control conditions (Fig. 4A1-3; Supplemental Fig. 3A). In contrast, robust C3 immunoreactivity was observed in GFAP-positive hypertrophic astrocytes at 3 weeks post-SE (Fig. 4B2i; Supplemental Fig. 3B), with a similar pattern also evident at 2 weeks post-SE (Supplemental Fig. 4). Images showing the xy, yz, and xz views further demonstrate that C3 localized predominantly within GFAP-positive astrocytic structures rather than DAPI-labeled nuclei. Representative high-magnification images are shown in Fig. 4, whereas low-magnification images are provided in Supplemental Fig. 3. These findings further corroborate and extend previous reports demonstrating astrocytic C3 expression under basal conditions and following SE, with expression emerging as early as 3–7 days post-SE and persisting for up to 5 weeks (Jeong et al. 2025, Kharatishvili et al. 2014, Jiang et al. 2025).

## Discussion

In this study, we characterized the temporal trajectory of GFAP in the hippocampus following pilocarpine-induced SE. Using complementary histological and biochemical approaches, we show that: (1) GFAP immunoreactivity increases in a time-dependent manner, peaking at 2–3 weeks and modestly declining by 5 weeks; (2) total GFAP protein levels measured by immunoblots remained significantly elevated from 2 to 5 weeks post-SE; (3) GFAP undergoes progressive proteolytic cleavage during epileptogenesis, and we quantitatively demonstrate increases in GFAP protein breakdown products, and (4) hypertrophic GFAP-positive astrocytes colocalize with the complement component C3 at 2–3 weeks after SE. Together, these findings provide an integrated view of astrocytic structural remodeling and complement association post-SE.

Our histological findings are consistent with the extensive literature demonstrating reactive astrogliosis following SE (Vezzani et al. 2022, Bedner et al. 2024, Robel et al. 2015, Rossi et al. 2017, Gomez Cuautle et al. 2026). Numerous studies have reported astrocyte hypertrophy, proliferation, and increased GFAP expression in the hippocampus within days to weeks after SE (Vezzani et al. 2022, Bedner et al. 2024, Shapiro et al. 2008, Borges et al. 2003, Arisi et al. 2011, Kim et al. 2008, Loewen et al. 2016, Estrada et al. 2012, Sano et al. 2021). In agreement with prior reports, we observed that GFAP immunostaining increased progressively after SE, peaking between 2 and 3 weeks (Yang et al. 2022). Although the overall pattern of increased GFAP expression during the subacute phase is well established, some variability has been reported across models and time points. For example, Shapiro et al. (2008) examined GFAP immunoreactivity 1–5 days post-SE and found no significant changes in the hilus, CA1, or CA3 regions compared to controls (Shapiro et al. 2008). In contrast, Estrada et al. (2012) reported an initial increase in the number of GFAP-positive cells in the hippocampus that peaked at 4 weeks and declined by 8 weeks after SE (Estrada et al. 2012). Together, these findings highlight that while reactive astrogliosis is a consistent feature after SE, the precise temporal profile may vary depending on experimental conditions, brain region analyzed, and methodological approach.

Importantly, our immunoblot analyses extend these observations by demonstrating sustained increases in total GFAP protein levels from 2 to 5 weeks after SE. Although IHC suggested that the GFAP immunosignal was less robust at 5 weeks than at the 2- to 3-week peak, the immunoblots revealed that overall GFAP protein levels remained significantly elevated. This apparent discrepancy highlights methodological differences between the approaches, as IHC is used to assess spatial distribution, whereas immunoblots assess total protein content in hippocampal homogenates. The divergence between IHC and WB findings at 5 weeks raises an important mechanistic consideration. It is possible that cleavage of GFAP into smaller fragments alters filament organization or epitope availability, thereby reducing the apparent intensity or structural coherence of GFAP immunostaining despite sustained or elevated total protein levels. Such cleavage may disrupt intermediate filament assembly or redistribute GFAP fragments within astrocytes in ways that are not fully captured by DAB-based IHC (Yang and Wang 2015). Although this interpretation remains speculative, it warrants further investigation using domain-specific GFAP antibodies or high-resolution imaging to assess filament integrity.

A particularly novel aspect of our study is the quantification of GFAP cleavage products after SE. While prior work has reported GFAP breakdown products under pathological conditions (Yang and Wang 2015, Mouser et al. 2006), these fragments have not been systematically quantified in the context of SE-induced epileptogenesis. We observed the emergence of cleaved GFAP fragments beginning at 3 days post-SE, with significant accumulation from 2 to 5 weeks. The concurrent elevation of full-length GFAP and its cleavage products suggests that astrocytes not only increase GFAP synthesis but also are undergoing active cytoskeletal remodeling and proteolytic processing post-SE.

GFAP is a crucial intermediate filament protein that supports the structural stability of astrocytes, and its cleavage has significant functional consequences. Under normal conditions, GFAP is a ~ 50-kDa intermediate filament protein essential for preserving astrocyte cytoskeletal integrity (Yang and Wang 2015). In pathological conditions, GFAP undergoes proteolytic cleavage, serving as a sensitive marker of cytoskeletal destabilization, astrocytic stress, or transitions toward degenerative phenotypes (Yang and Wang 2015, Mouser et al. 2006). Following neurotrauma or glial insult, GFAP is cleaved by calpains and caspases into smaller breakdown products (BDPs) that lack the N- and C-terminal regions necessary for filament assembly (Yang and Wang 2015, Yang et al. 2022, Zhang et al. 2014). GFAP-BDPs can appear within hours after injury and are reported to be more sensitive than full-length GFAP for detecting acute astrocyte pathology, potentially indicating a shift from reactive gliosis to astrocyte degeneration (Yang and Wang 2015, Yang et al. 2022, Zhang et al. 2014, Yang et al. 2018, Abdelhak et al. 2022). These fragments can be released into the extracellular space, CSF, and bloodstream through a leaky BBB, and may become immunogenic, possibly triggering autoimmune responses against astroglia. While previous work in SE and epilepsy models, including our own, has detected GFAP cleavage products on immunoblots (Robel et al. 2015, Schartz et al. 2024, Wyatt-Johnson et al. 2021), none had quantified these fragments. We observed the emergence of cleaved GFAP fragments beginning at 3 days post-SE, with significant accumulation from 2 to 5 weeks. The simultaneous increase in full-length GFAP and its cleavage products indicates that astrocytes are not only producing more GFAP but also actively remodeling their cytoskeleton and undergoing proteolytic processing during epileptogenesis. Therefore, this buildup of GFAP fragments may indicate ongoing astrocytic remodeling, changes in mechanical properties, or even impaired astrocyte function. In addition, circulating GFAP BDPs have been suggested as sensitive biomarkers of astroglial injury in other neurological disorders (Yang and Wang 2015, Yang et al. 2022, Zhang et al. 2014, Gogishvili et al. 2025). Our findings suggest they may serve as biomarkers of astrocytic reactivity post-SE, with potential translational value for tracking disease progression.

Importantly, astroglial responses during epileptogenesis extend beyond structural changes in GFAP expression and cleavage. Recent evidence indicates that astrocytes undergo broad transcriptional and epigenetic reprogramming during the epileptogenic period, characterized by repression of homeostatic genes and activation of proinflammatory programs through DNA methylation–dependent mechanisms (Gomez Cuautle et al. 2026). These changes include downregulation of key astrocytic homeostatic genes and induction of inflammatory gene networks, suggesting a stable shift in astrocyte functional state that is not captured by GFAP-based analyses alone (Gomez Cuautle et al. 2026).

Another key finding of this study is the colocalization of GFAP with complement component C3 in hypertrophic astrocytes. We observed a robust C3 signal in GFAP-positive astrocytes during the 2- to 3-week period after SE, temporally aligning with peak GFAP upregulation. While we were preparing this manuscript, two recent studies independently reported astrocytic C3 upregulation at different time points following SE in experimental models and in human TLE brain biopsies (Jeong et al. 2025, Jiang et al. 2025), further supporting the reproducibility of this observation. Despite converging evidence for astrocytic C3 expression, the functional consequences of this colocalization remain unclear. Whether C3 expression in astrocytes following SE promotes maladaptive synaptic pruning, alters neuronal excitability, or instead represents a compensatory response requires further mechanistic studies. Notably, SE-induced increases in astrocytic C3 expression in the hippocampus have been reported to correlate with increased spike frequency during epileptogenesis (Kharatishvili et al. 2014). Additionally, we found that C3 knockout mice did not exhibit SE-induced increases in hippocampal GFAP, supporting a role for C3 in SE-induced astrocyte changes at two weeks post-SE (Schartz et al. 2023). However, C3 may also be produced by and signal through other cell types, including microglia (Wei et al. 2020) and endothelial cells (Propson et al. 2021). Here, we focused on astrocytic C3, and future studies are needed to determine its broader cellular sources and the signaling pathways it activates after SE.

Astrocyte responses following SE are dynamic and may become maladaptive, potentially contributing to the onset and progression of acquired epilepsy (Vezzani et al. 2022, Bedner et al. 2024, Rossi et al. 2017, Gomez Cuautle et al. 2026). Increasing evidence indicates that interactions between astrocytes and other cell types, particularly microglia and neurons, play a critical role in shaping reactive astrogliosis following SE (Vezzani et al. 2022, Bedner et al. 2024, Rossi et al. 2017). In our time-course analysis integrating astrocytic, microglial, and neuronal/dendritic markers, we found that microglial activation begins within hours after SE and peaks at approximately 2 weeks (Wyatt-Johnson et al. 2017, Schartz et al. 2016), whereas GFAP immunoreactivity, as measured by immunoblotting, persists beyond this time point. This temporal sequence is consistent with prior reports, in which reactive microglia appear first in the CA1 hippocampus, followed by reactive astrogliosis, a progression associated with increased seizure susceptibility (Sano et al. 2021). One potential mechanism underlying this coordinated response is microglia-astrocyte communication via complement signaling, including C1q and C3 signaling (Wei et al. 2020). This C1q-C3 pathway parallels the SE-induced trajectory of GFAP upregulation observed in our study (Schartz et al. 2024, Schartz et al. 2019, Schartz et al. 2018), supporting the possibility that complement-mediated crosstalk contributes to sustained astrocytic reactivity during epileptogenesis and the emergence of seizure activity. However, the lack of concurrent EEG recordings prevents us from directly relating GFAP dynamics or complement-associated astrocytic changes to electrographic seizure activity, including seizures or fast ripples, which are linked to gliosis (Chipres-Tinajero et al. 2021).

In summary, we provide a comprehensive temporal characterization of astrocytic GFAP dynamics following SE using complementary histological and biochemical approaches. We replicate the well-established peak in GFAP immunoreactivity at 2–3 weeks post-SE (Schartz et al. 2024, Schartz et al. 2018, Wyatt-Johnson et al. 2017, Schartz et al. 2016, Brewster et al. 2013, Wyatt-Johnson et al. 2021), demonstrate sustained elevation of total GFAP protein through 5 weeks, and quantitatively document progressive GFAP cleavage during the latent period post-SE. The colocalization of GFAP with C3 further implicates complement-associated astrocytic responses in this process. Together, these findings further demonstrate the dynamic and multifaceted nature of astrocyte remodeling after SE and highlight GFAP synthesis and cleavage as coordinated components of the astrocytic response post-SE.

## Supplementary Information

Below is the link to the electronic supplementary material.

Supplementary Material 1

## Data Availability

The data that support the findings of this study are available from the corresponding author upon reasonable request.
