# Supplementary material for: Temporal profiling of hippocampal astrocyte reactivity reveals persistent GFAP cleavage and complement C3 association after status epilepticus
Source: Brain Struct Funct. 2026 Aug 7;231(7):116. doi: 10.1007/s00429-026-03175-9 (PMC13451406; doi:10.1007/s00429-026-03175-9)
Supplement: Supplementary file 1 [file 429_2026_3175_MOESM1_ESM.pdf]

Supplemental Figures

Title: Temporal profiling of hippocampal astrocyte reactivity reveals persistent GFAP cleavage and complement C3 association after status epilepticus

Author list:
Alisha Aroor[1#]
Zahra Sadri[2#]
Nicole Pinzón-Hoyos [2]
Nicole D. Schartz[1]
Amy L. Brewster[*1,2]

Affiliations:
[1] Department of Psychological Sciences, Purdue University, West Lafayette, IN 47907.
[2] Department of Biological Sciences, Southern Methodist University, Dallas, TX 75205.

[#]Authors contributed equally

*Corresponding author:

Amy L. Brewster, Ph.D.
Associate Professor
Department of Biological Sciences
Dedman College of Humanities and Sciences
Southern Methodist University
6501 Airline Rd, Dallas, TX 75205
Office: DLSB 240; Lab DLSB 215/219
Tel: 214-768-5949
Email: albrewster@smu.edu

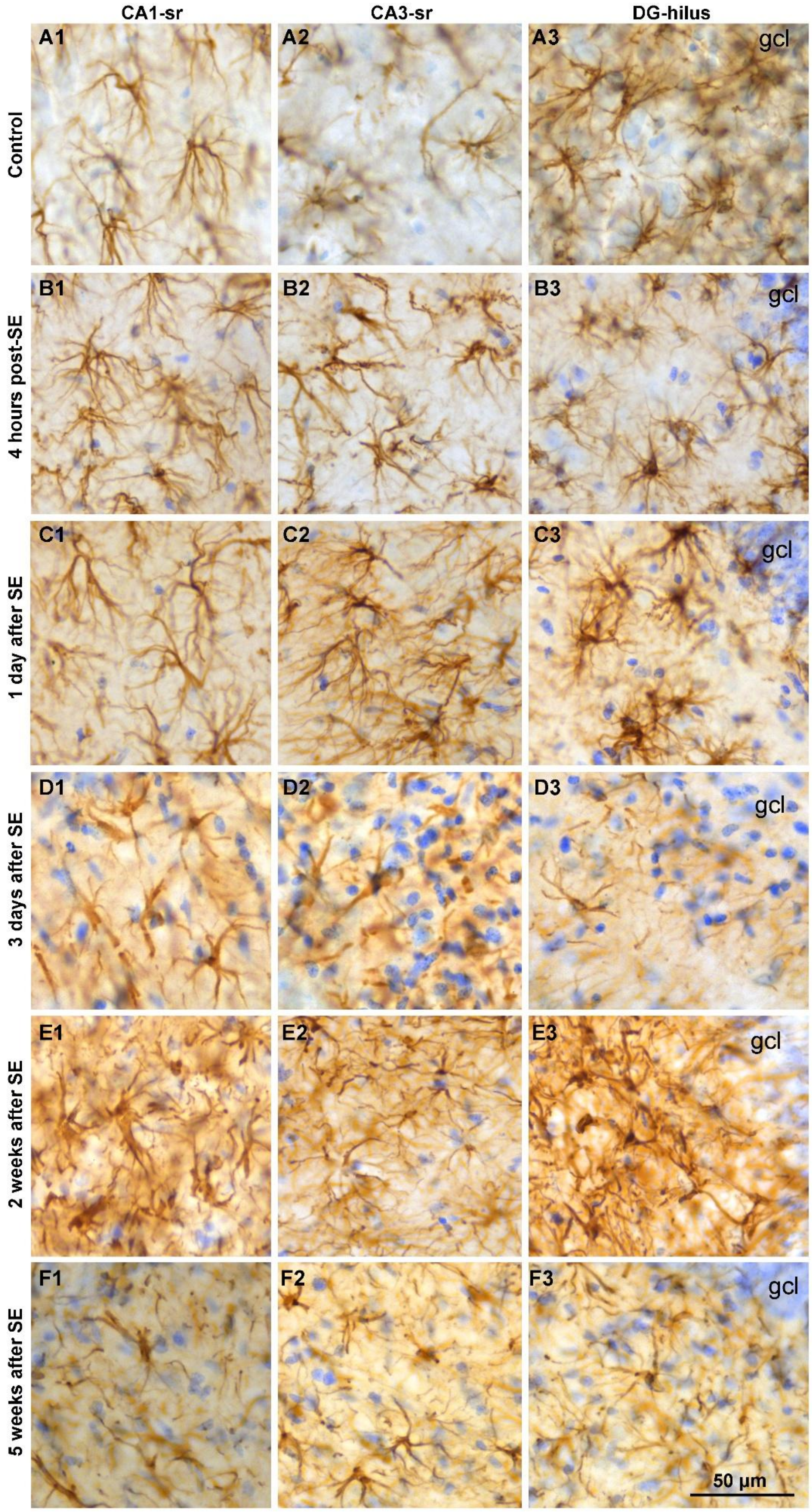

Supplemental Figure 1. Temporal changes in GFAP immunoreactivity in the CA1, CA3, and DG areas of the hippocampus following status epilepticus (SE).

Supplemental Figure 1. Temporal changes in GFAP immunoreactivity in the CA1, CA3, and DG areas of the hippocampus following status epilepticus (SE). Representative high magnification photomicrographs showing GFAP-positive astrocytes (brown staining) in CA1, CA3, and dentate gyrus areas of the hippocampus from control animals and at different time points after SE. Columns represent hippocampal subregions: CA1 stratum radiatum (CA1-sr; A1-F1), CA3 stratum radiatum (CA3-sr; A2-F2), and dentate gyrus hilus (DG-hilus; A3-F3). Rows correspond to experimental time points: control (A1-A3), 4 h after SE (B1-B3), 1 day after SE (C1-C3), 3 days after SE (D1-D3), 2 weeks after SE (E1-E3), and 5 weeks after SE (F1-F3). Compared with controls, astrocytes exhibited progressive reactive gliosis after SE, characterized by hypertrophy of cell bodies, thickening and elongation of processes, and increased GFAP immunoreactivity, most pronounced at 2 weeks post-SE. Partial attenuation of reactive changes was observed at 5 weeks after SE. Nuclei were counterstained with Nissl stain (blue). Scale bar = 50 µm.

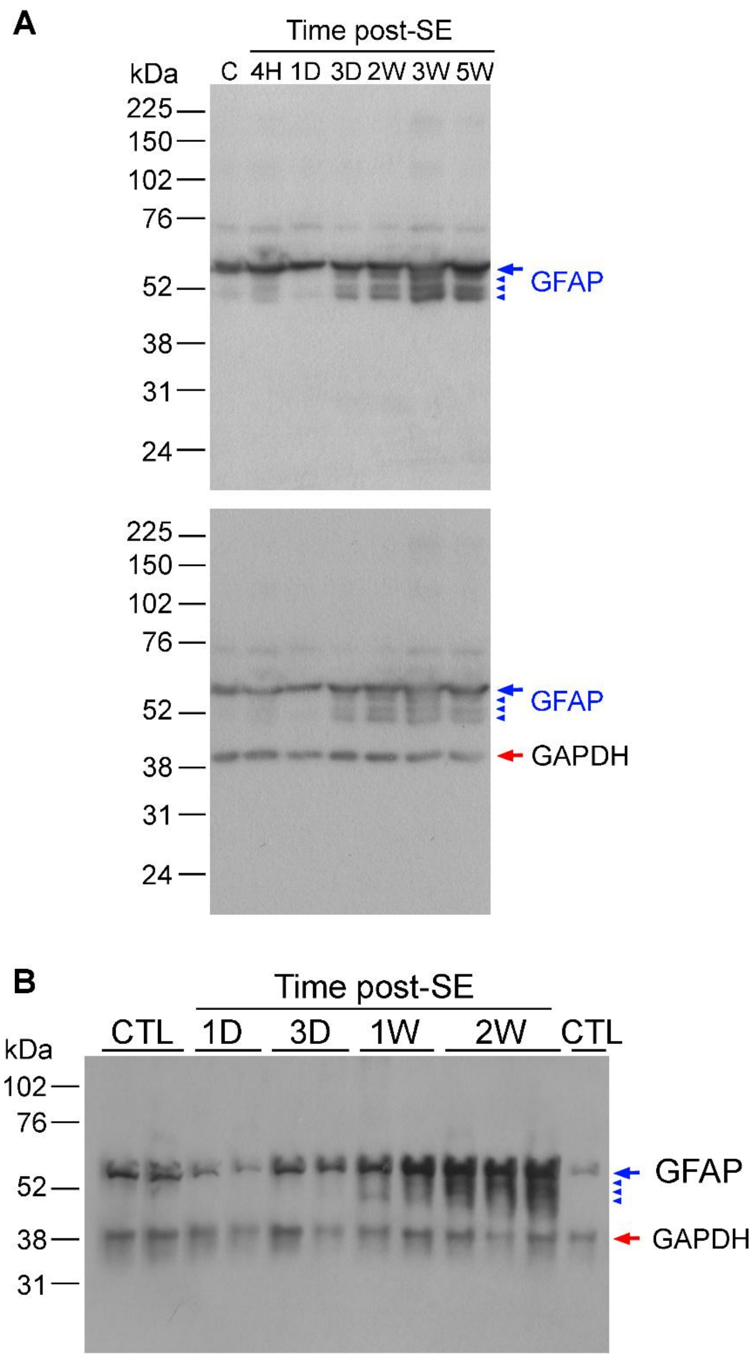

Supplemental Figure 2. Temporal profile of GFAP protein levels and cleavage in the hippocampus following status epilepticus (SE). (A) Representative western blots showing GFAP immunoreactivity in hippocampal homogenates from control (C) rats and rats at 4 hours (4H), 1 day (1D), 3 days (3D), 2 weeks (2W), 3 weeks (3W), and 5 weeks (5W) post-SE. The upper panel shows GFAP bands, including the full-length protein (~50 kDa) (Blue arrow) and lower-molecular-weight cleavage fragments (arrowheads). The lower panel shows GFAP with GAPDH (red arrow) as a loading control (uncropped). GAPDH antibodies were added after GFAP immunoblotting was completed. Molecular weight markers (kDa) are indicated on the left. **(B)** Additional representative blot illustrating GFAP expression in control (CTL) and post-SE time points (1D, 3D, 1W, 2W), with GAPDH as loading control. Increased full-length GFAP and accumulation of cleavage products are evident at later time points after SE.

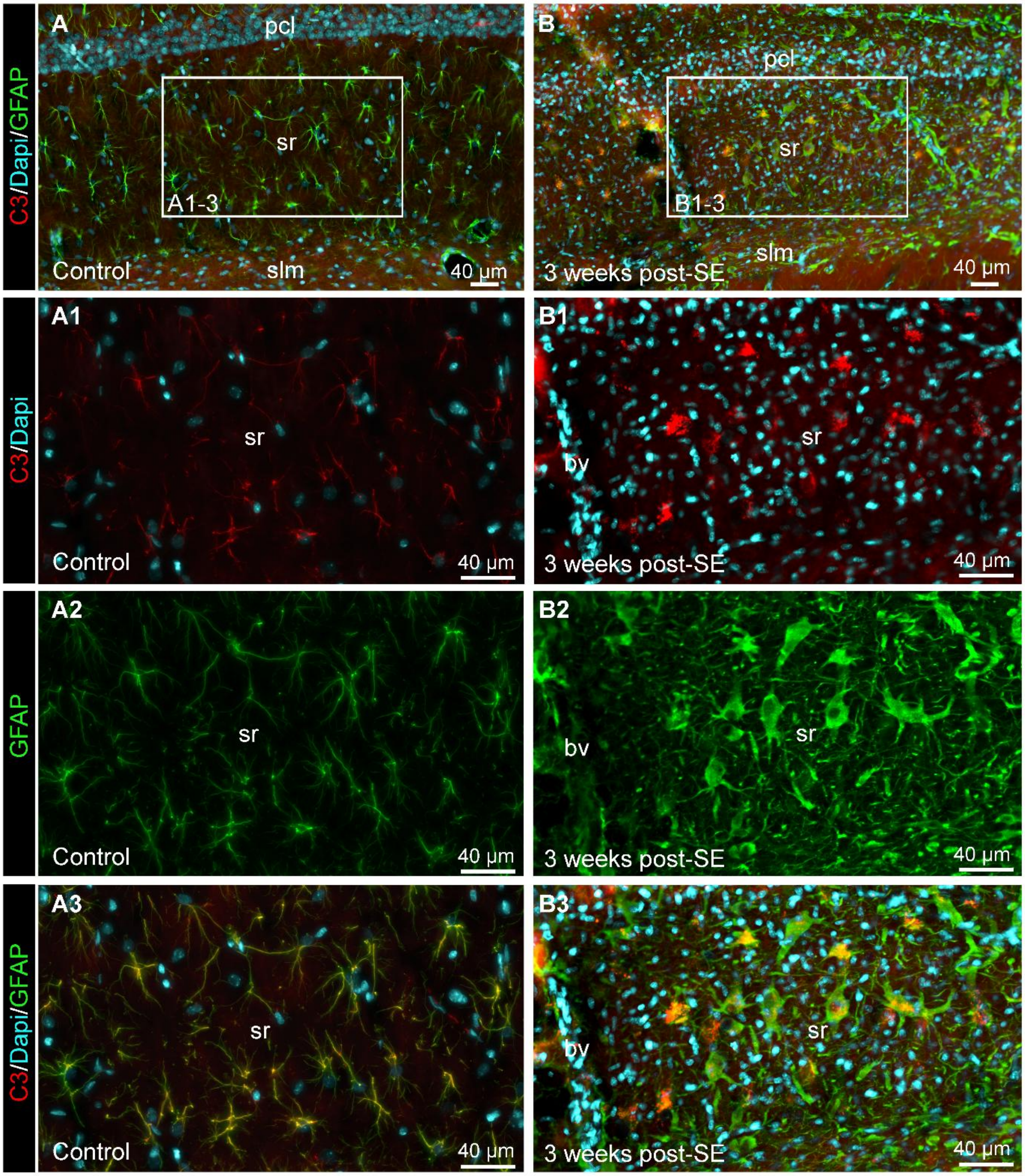

Supplemental Figure 3. Colocalization of GFAP and C3 in the hippocampal CA1 region following status epilepticus (SE). (A-B) Representative low-magnification immunofluorescence images of the CA1 hippocampal region from control rats (A) and rats at 3 weeks post-SE (B), stained for C3 (red), GFAP (green), and DAPI (blue). Boxed areas indicate the stratum radiatum (sr) regions shown at higher magnification in panels A1-A3 and B1-B3. Abbreviations: pcl, pyramidal cell layer; sr, stratum radiatum; slm, stratum lacunosum-moleculare; bv, blood vessel. (A1-A3) Higher-magnification images from a control hippocampus showing C3 (A1), GFAP (A2), and merged C3/GFAP/DAPI (A3) channels in the sr. In controls, GFAP-positive astrocytes display characteristic stellate morphology. (B1-B3) Higher-magnification images from 3 weeks post-SE hippocampus showing C3 (B1), GFAP (B2), and merged C3/GFAP/DAPI (B3) channels in the sr. At 3 weeks post-SE, hypertrophic GFAP-positive astrocytes exhibit strong C3 immunoreactivity, with clear colocalization evident in the merged images (yellow signal). Scale bars as indicated.

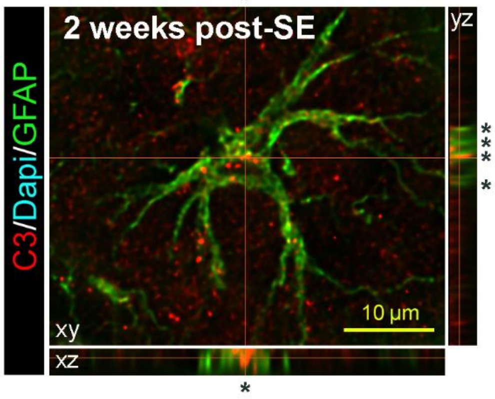

Supplemental Figure 4. Colocalization of GFAP and C3 in the hippocampal CA1 stratum radiatum region 2 weeks following status epilepticus (SE). Representative images of hippocampal tissue from a rat 2 weeks after SE induction. XY, XZ, and YZ image projections showing C3 immunoreactivity (red) in relation to cell GFAP (green). Representative C3-positive structures colocalize with GFAP. Asterisks denote regions of signal overlap.
